# Thrips detection on sticky traps based on improved YOLOv11n

**DOI:** 10.3389/fpls.2026.1945602

**Published:** 2026-10-29

**Authors:** Shuxing Zhou, Yang Li, Yuheng Lu, Haoyang Liu, Jun Li, Yuhan Pan, Xiaowei Li, Jinming Zhang, Zhijun Zhang, Jun Huang, Wanying Dong, Yaobin Lu

**Affiliations:** 1 State Key Laboratory for Quality and Safety of Agro-products, Xianghu Laboratory, Institute of Plant Protection and Microbiology, Zhejiang Academy of Agricultural Sciences, Hangzhou, China; 2 Key Laboratory of Tea Quality and Safety Control, Ministry of Agriculture and Rural Affairs, Tea Research Institute, Chinese Academy of Agricultural Sciences, Hangzhou, China; 3 Hangzhou Ruikun Technology Co., Ltd., Hangzhou, China; 4 Anji County Agriculture and Rural Affairs Bureau, Huzhou, China

**Keywords:** EfficientHead, normalized Wasserstein distance (NWD), thrips detection, tiny object detection, wavelet unpooling StarFusion (WSF), YOLOv11n

## Abstract

Thrips on sticky traps are extremely small and densely distributed, making automated detection challenging. This study proposes YOLOv11n-WNE, an improved YOLOv11n-based detector tailored for thrips detection in greenhouse environments. Three modifications are introduced: (1) a Wavelet Unpooling StarFusion (WSF) network in the neck, combining WaveletUnPool upsampling—which uses the discrete wavelet transform to preserve spatial details—with StarFusion feature enhancement that captures high-order multi-scale interactions through element-wise multiplication; (2) a Normalized Wasserstein Distance (NWD) loss that models bounding boxes as 2-D Gaussian distributions, providing smooth gradient responses for tiny-object localization; and (3) an EfficientHead detection head using grouped convolutions (G = 8) to compress parameters and computation. A dataset of 2, 400 images with 40, 480 annotated thrips instances was constructed for training (1, 680), validation (360), and testing (360). YOLOv11n-WNE achieves mAP@0.5 of 91.5%, with precision of 88.2% and recall of 85.0%—improvements of 4.1, 4.5, and 5.1 percentage points over the baseline YOLOv11n, respectively—while reducing parameters to 2.11M (81.8% of baseline) and model size to 4.5 MB. On the GPU, the model runs at 123.8 FPS at an input size of 640×640, supporting its deployment for edge-based intelligent thrips monitoring in greenhouses. Beyond individual novelty, the contribution lies in problem-driven integration. WSF, NWD, and EfficientHead act on complementary stages of the detection pipeline—feature aggregation, localization supervision, and model compression, respectively. Together they address the failure modes of thrips-on-sticky-trap imagery: dense tiny objects, weak textures, and frequent overlap with non-target insects.

## 1 Introduction

Thrips (order Thysanoptera) are minute insects whose adults generally measure only a few millimetres or less (He et al., 2020). Across global agroecosystems they rank among the most economically destructive groups of insect pests. Of the more than 6000 described species, over 100 function as plant pests in agricultural, horticultural, and forestry settings; prominent examples include *Frankliniella occidentalis, Frankliniella intonsa, Megalurothrips usitatus*, and *Thrips tabaci*. China possesses the largest protected-agriculture area in the world, with greenhouse cultivation exceeding 4 million hm^2^, playing an irreplaceable role in securing the “vegetable basket” supply and increasing farmers income (Nie et al., 2024). However, the high-temperature and high-humidity enclosed environment of greenhouses readily fosters pest proliferation, posing a serious threat to crop yield and quality. Both adult and larval stages of phytophagous thrips damage plants by piercing leaves, flowers, and fruits with their stylets and draining cellular fluids; this injury lowers photosynthetic capacity, suppresses growth and reproduction, and ultimately imposes heavy economic losses. In addition to direct feeding injury, thrips serve as vectors for plant pathogens such as tospoviruses (e.g., tomato spotted wilt virus, TSWV), further exacerbating losses (Kang et al., 2025; Rotenberg and Whitfield, 2018; Wang et al., 2026).

Several biological attributes render phytophagous thrips especially prone to successful invasion and outbreak: their small body size, cryptic habits, high reproductive output, and strong dispersal capacity (He et al., 2020; Reitz et al., 2011). Once a population becomes established, certain species can proliferate rapidly and attain outbreak densities within a short period, complicating management and inflicting substantial economic damage. At present, greenhouse pest monitoring commonly relies on yellow or blue sticky traps as trapping media, with thrips counted and identified manually to assess population dynamics (Niyigena et al., 2023). Nevertheless, manual counting suffers from low efficiency, heavy labor intensity, and strong subjectivity. Because thrips are extremely small and difficult to distinguish from background debris on sticky traps, prolonged counting causes visual fatigue and leads to missed and false detections. When pest density is high, overlapping and adherent individuals further increase the difficulty of counting (Ghosh et al., 2021). Moreover, sticky-trap images are typically captured at high resolution, yet thrips occupy an extremely low proportion of pixels (usually only 0.001%-0.01% of the total), constituting a typical tiny-object detection scenario (Meng et al., 2026). Reliable monitoring of thrips populations is therefore an indispensable component of integrated pest management, as it permits the early detection of incursions and supports the timely deployment of control tactics before economically meaningful losses accumulate. There is thus an urgent need to develop automated, intelligent thrips detection and counting methods to replace traditional manual monitoring.

In recent years, deep-learning-based object detection has shown great potential in agricultural pest recognition. Existing methods are mainly divided into two-stage and one-stage detectors. Two-stage detectors first generate region proposals and then classify and regress them. Typical two-stage detectors include R-CNN, Fast R-CNN, and Faster R-CNN (Girshick, 2015; Girshick et al., 2014; Ren et al., 2017). In agricultural pest detection, Li et al. (2021) proposed the TPest-RCNN model for greenhouse pest classification, achieving a validation F1-score of 0.944. However, two-stage methods entail heavy computation and slow inference, making them difficult to satisfy real-time requirements on edge devices.

One-stage detectors directly predict object categories and bounding-box positions through an end-to-end network, featuring low computational cost and fast inference, and are thus more suitable for practical deployment. The YOLO (You Only Look Once) family is a representative one-stage detector (Jocher et al., 2024; Redmon and Farhadi, 2018; Redmon et al., 2016; Wang et al., 2023). For sticky-trap pest detection, Huang et al. (2025) proposed YOLO-YSTs, reaching mAP@0.5 of 86.8% at 139 frames/s on yellow sticky traps; and Meng et al. (2026) proposed a sticky-trap pest detection method fusing SAHI slice inference with the YOLOv11n-LMN model, lifting mAP@0.5 to 91.3% after introducing a PP-LCNet lightweight backbone, a MulSimAM attention mechanism, and the NWD loss.

In a closely related work, Zeng et al. (2025) proposed a lightweight tea pest and disease recognition method based on improved YOLOv11n, employing a WaveletUnPool–StarFusion (WSF) network in the neck and an EfficientHead detection head with grouped convolutions. While their work demonstrates the effectiveness of these modules for agricultural pest detection, it targets tea diseases with relatively larger lesion areas and does not address the challenges of extremely small, densely distributed objects such as thrips on sticky traps. Moreover, their approach does not incorporate a specialized small-object regression loss such as NWD, which is critical for pixel-level localization of tiny pests. The present study adopts the WSF network and EfficientHead in a different application context and, more importantly, contributes a NWD-optimized joint loss strategy specifically designed for thrips-scale objects, together with a dedicated thrips dataset.

Despite this progress, sticky-trap thrips detection still faces the following challenges: (1) thrips are tiny objects (typically only 10–40 pixels in an image), and existing models still fall short in small-object accuracy; (2) the parameter count and computation of existing models can challenge efficient operation on resource-constrained edge devices; (3) information loss during upsampling in the neck network is severe, impairing the preservation and reconstruction of small-object spatial details; and (4) traditional detection heads contain redundant computation, adding unnecessary resource overhead.

In this work, we propose YOLOv11n-WNE for small, densely overlapping thrips detection. The method integrates a Wavelet Unpooling StarFusion (WSF) neck to improve feature reconstruction of tiny thrips, adopts the Normalized Wasserstein Distance (NWD) loss to enhance the localization of small, densely overlapping instances, replaces the detection head with a lightweight EfficientHead based on group convolution to reduce model complexity, and is trained and evaluated on a dedicated thrips detection dataset constructed with image-slicing preprocessing.

## 2 Materials and methods

### 2.1 Dataset construction and preprocessing

#### 2.1.1 Data collection

To construct a high-quality thrips detection dataset and to approximate real-world application scenarios so as to improve model performance, this study manually screened clear images of thrips on sticky traps for training the object-detection and counting model. Data acquisition included thrips at various developmental stages (nymphs and adults). Acquisition devices included an agricultural IoT pest-monitoring instrument and smartphones, with image resolutions ranging from 3000×4000 to 4032×3024 pixels. After image slicing and manual screening, a total of 40,480 thrips instances were annotated in the dataset, covering all life stages of thrips on sticky traps, which ensures dataset diversity and the generalizability of the model.

#### 2.1.2 Data preprocessing

A total of 1, 000 original high-resolution sticky-trap images were acquired from greenhouse fields; each image had a resolution no lower than 3000×4000 pixels and contained dozens to hundreds of thrips individuals scattered across the trap surface.

Because the original images are of high resolution (no lower than 3000×4000 pixels) while thrips occupy only a very small proportion of each image (typically 10–40 pixels), directly resizing the original images to the model input size (640×640 pixels) would cause severe loss of thrips detail and degrade detection accuracy. To improve small-object detection, the original images were divided into equal-sized 640×640 pixel sub-images, thereby increasing the pixel proportion of thrips and enhancing small-object detection performance. An overlap ratio of 0.1 was set between adjacent slices to preserve object integrity. Through slice preprocessing, the pixel proportion of thrips in the sub-images increased, which facilitates the model’s learning of small-object features. Only crops containing at least one annotated thrips instance were retained; this yielded 2, 400 thrips-containing 640×640 sub-images (Section 3.1).

After slicing the original high-resolution images into 640×640 sub-images and screening, a total of 2, 400 thrips-containing images were obtained, with 1, 680 for training, 360 for validation, and 360 for testing. The training set contains approximately 28, 336 annotated thrips instances, the validation set approximately 6, 072 instances, and the test set approximately 6, 072 instances. In addition, data augmentation strategies such as Mosaic augmentation, random flipping, and HSV color-space perturbation were applied during training to further improve the model’s generalization and robustness.

Dataset annotation was performed with the LabelImg tool. All thrips objects were precisely annotated with rectangular bounding boxes in the YOLO format (class ID + normalized center coordinates + normalized width and height). The dataset split and annotation statistics are summarized in **Table 1**.

**Table 1:** Dataset split and annotation statistics.

Dataset split	Number of images	Number of thrips annotations
Training set	1680	28336
Validation set	360	6072
Test set	360	6072
Total	2400	40480

### 2.2 Improved YOLOv11n detection model (YOLOv11n-WNE)

#### 2.2.1 YOLOv11n baseline model overview

Released by the Ultralytics team in 2024, YOLOv11n follows the end-to-end one-stage detection architecture of the YOLO series and consists of three components: the backbone, the neck, and the head (Jocher et al., 2024). The backbone adopts a CSPDarknet variant, extracting multi-scale features through the C3k2 and C2PSA (Cross-stage Partial Spatial Attention) modules and expanding the receptive field with the SPPF (Spatial Pyramid Pooling–Fast) module. The neck adopts a PAN-FPN (Path Aggregation Network–Feature Pyramid Network) structure, performing multi-scale feature fusion through top-down and bottom-up bidirectional paths. The head uses a decoupled structure that separately predicts object category and bounding-box location.

As the lightest variant of the YOLOv11 family, YOLOv11n has a model size of only 5.5 MB (fp32) and achieves an mAP@0.5 of 59.7% on the COCO dataset (Jocher et al., 2024), striking a good balance between accuracy and speed, which makes it a popular choice for edge-device deployment. However, YOLOv11n still faces the following shortcomings in the sticky-trap thrips detection task: (1) the nearest-neighbor upsampling in the neck cannot effectively preserve the spatial details of small objects during feature reconstruction, which is detrimental to precise thrips localization; (2) the CIoU loss is overly sensitive to small-object position deviations, making it difficult for extremely small objects such as thrips to match positive samples and thereby hurting recall; and (3) the standard convolution (groups = 1) used in the head performs dense connections across feature channels, incurring redundant computational cost.

The choice of YOLOv11n as the baseline, rather than the adjacent YOLOv10n (Wang et al., 2024) or YOLOv12n (Tian et al., 2025) releases, was based on three considerations. First, architectural suitability for tiny objects: the C3k2 module provides stable gradient paths through its two-branch aggregation structure, and the C2PSA spatial-attention module strengthens the representation of small, low-contrast targets, whereas the region-attention design of YOLOv12n targets larger-context modeling at a higher parameter cost, and the NMS-free dual-assignment head of YOLOv10n mainly benefits scenarios with many object classes rather than single-class tiny-object tasks. Second, efficiency: among the nano-scale variants, YOLOv11n offers a favorable accuracy–complexity balance for edge deployment. Third, maturity and reproducibility: YOLOv11n is maintained within the Ultralytics framework, which allows all compared models to be trained under identical hyperparameters for a fair comparison. Comparative results with YOLOv10n and YOLOv12n on the thrips dataset are provided in **Table 2**.

**Table 2:** Performance comparison with mainstream detection models.

Model	P (%)	R (%)	mAP@0.5 (%)	mAP@0.5:0.95 (%)	Params (M)	GFLOPs	Model size (MB)	FPS (GPU)	FPS (CPU)
RT-DETR	75.3	64.7	70.7	42.3	32.0	82.0	66.2	28.3	1.4
YOLOv5s	82.6	78.2	87.0	56.8	7.02	15.8	14.4	138.6	12.8
YOLOv7-tiny	72.1	61.2	66.8	38.5	6.02	13.0	11.7	162.4	22.7
YOLOv8n	83.7	77.3	86.2	54.1	3.01	8.1	6.2	188.5	21.5
YOLOv11n	83.7	79.9	87.4	55.6	2.58	6.3	5.5	165.0	19.6
YOLOv10n	81.4	77.5	86.1	54.7	2.30	6.7	4.9	172.4	20.1
YOLOv12n	84.2	80.6	88.0	56.5	2.56	6.5	5.2	153.7	17.8
YOLOv5n+TPH	80.5	76.8	84.3	53.6	5.50	14.2	11.5	98.6	11.2
YOLOv11n-BiFPN	84.3	80.8	88.2	56.9	2.42	6.6	5.0	142.6	16.4
YOLOv11n-WNE (ours)	88.2	85.0	91.5	60.2	2.11	6.0	4.5	123.8	14.5

To address the above issues, the improved model proposed in this paper is named YOLOv11n-WNE (WSF + NWD + EfficientHead), and its overall architecture is shown in **Figure 1**. Three key improvements are introduced: the WSF module replaces the original upsampling and feature-fusion structure in the neck to enhance small-object feature representation; the NWD loss is introduced into the bounding-box regression branch to improve localization accuracy; and the EfficientHead replaces the original decoupled head in the detection head to achieve lightweighting.

**Figure 1:**
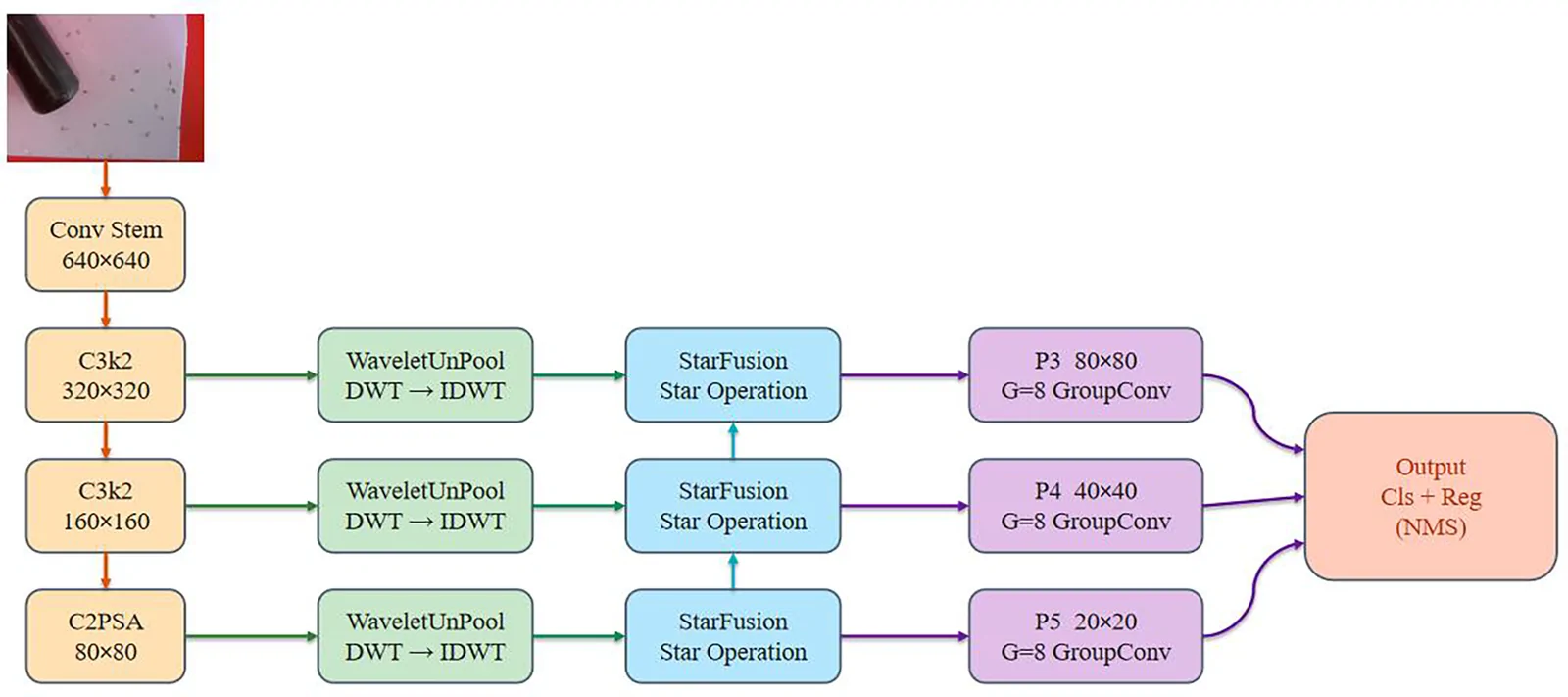
Overall network architecture of YOLOv11n-WNE.

#### 2.2.2 WSF: wavelet unpooling StarFusion network

##### 2.2.2.1 WaveletUnPool wavelet-based upsampling

In traditional YOLO neck networks, feature upsampling is typically implemented by nearest-neighbor interpolation or transposed convolution. Nearest-neighbor interpolation enlarges features by directly copying adjacent pixel values; although computationally simple, it causes a “checkerboard” effect on the feature map and loses substantial spatial detail (Shi et al., 2016). Transposed convolution, while having learnable parameters, introduces extra computational overhead and may produce uneven overlap effects. For extremely small objects such as thrips that occupy only a few pixels on the feature map, information loss during upsampling will seriously affect the subsequent head’s precise localization of objects.

To overcome the above problems, this paper proposes the WaveletUnPool wavelet-based upsampling module. It uses the two-dimensional discrete wavelet transform (2D-DWT) to decompose the original feature map into four sub-band components, and then reconstructs the upsampled feature via the inverse discrete wavelet transform (2D-IDWT), preserving high-frequency detail while retaining low-frequency global information.

Specifically, given an input feature map F ∈ R^{C×H×W}, a Haar wavelet basis is used to perform 2D-DWT, decomposing it into four sub-band components:


F_LL, F_LH, F_HL, F_HH=DWT_Haar(F)

where F_LL is the low-frequency approximation component that preserves the overall structure of the original features; F_LH, F_HL, and F_HH are the high-frequency detail components in the horizontal, vertical, and diagonal directions, respectively, encoding spatial structural information such as edges and textures. Each sub-band has a resolution of H/2 × W/2 and C channels.

After feature processing, the four sub-band components are reconstructed into a feature map with doubled spatial resolution via the Haar inverse discrete wavelet transform, as shown in Equation 1:

(1) F_up=DWT_Haar(F′_LL, F′_LH, F′_HL, F′_HH)∈R^{C×2H×2W}

Because the high-frequency detail components are preserved during transformation and reconstruction, WaveletUnPool can effectively recover object edges and texture information after upsampling, significantly reducing the loss of small-object spatial details compared with nearest-neighbor interpolation. Furthermore, the Haar wavelet transform involves only addition and subtraction operations, with computational complexity O(N) and no additional learnable parameters, balancing computational efficiency and feature fidelity.

##### 2.2.2.2 StarFusion feature enhancement module

In multi-scale object detection, feature maps at different scales contain semantic information of different granularity: shallow features have high spatial resolution and rich detail but insufficient semantic information, whereas deep features have strong semantic information but low spatial resolution and blurred detail. Traditional feature-fusion methods (e.g., element-wise addition or channel concatenation) can only achieve a linear combination of features and struggle to capture the non-linear high-order interactions among features at different scales (Liu et al., 2018; Ma et al., 2024).

Inspired by the star operation idea in StarNet (Ma et al., 2024), this paper designs the StarFusion star-shaped feature-enhancement module. Through a multi-branch parallel structure, it performs transform projections of the input feature with different receptive fields, and then uses element-wise multiplication (star operation) to capture the multiplicative interaction among features of different branches, thereby mining the high-order semantic associations among multi-scale features.

The detailed structure of the StarFusion module is as follows: given an input feature map X ∈ R^{C×H×W}, a 1×1 convolution first compresses the number of channels to C/r (r is the reduction ratio, set to r = 4 in this paper) to reduce computational cost. The compressed feature is then processed through three parallel branches:

Branch 1 (identity mapping): directly retains the compressed feature X′ (C/r channels) to ensure information integrity.

Branch 2 (3×3 depthwise separable convolution): captures local spatial context through a 3×3 depthwise separable convolution, enhancing the neighborhood feature representation of small objects.

Branch 3 (5×5 depthwise separable convolution): enlarges the receptive field through a 5×5 depthwise separable convolution to capture spatial dependencies over a larger range.

The outputs of the three branches (denoted X_1_, X_2_, X_3_) interact through the star operation via element-wise multiplication, as shown in Equation 2:

(2) $$\text{F\_star}={\text{X}}_{1}⊙{\text{X}}_{2}⊙{\text{X}}_{3}$$

where ⊙ denotes element-wise multiplication (Hadamard product). The star operation implicitly establishes a high-order non-linear relationship in the feature space through multiplicative interaction, which can effectively enhance the cooperative representation ability among features at different scales. Studies (Chen et al., 2022; Ma et al., 2024) show that this element-wise multiplicative interaction can implicitly expand the feature dimension in an exponential space, thereby obtaining stronger representation ability at a very low computational cost.

Subsequently, a channel-attention mechanism is applied to the star-interaction result for adaptive weighting, as shown in Equation 3:

(3) $$\text{F\_att}=\sigma \left({\text{W}}_{2}\cdot \delta \left({\text{W}}_{1}\cdot \text{GAP}\left(\text{F\_star}\right)\right)\right)\cdot \text{F\_star}$$

where GAP is global average pooling, W_1_ and W_2_ are fully connected layer weights, δ is the ReLU activation function, and σ is the Sigmoid activation function. The channel-attention mechanism adaptively assigns importance weights to different channels, suppressing noisy channels and enhancing the responses of useful channels. Finally, a 1×1 convolution restores the number of feature channels to C, and a residual connection is made with the original input, as shown in Equation 4:

(4) F_out=Conv_{1×1}(F_att)/X

The StarFusion module is embedded at each feature-fusion node of the PAN-FPN in the neck, performing star-shaped fusion enhancement of multi-scale features after WaveletUnPool upsampling, so that the network obtains stronger semantic representation while preserving spatial details. The overall structure of the WSF network is shown in **Figure 2**.

**Figure 2:**
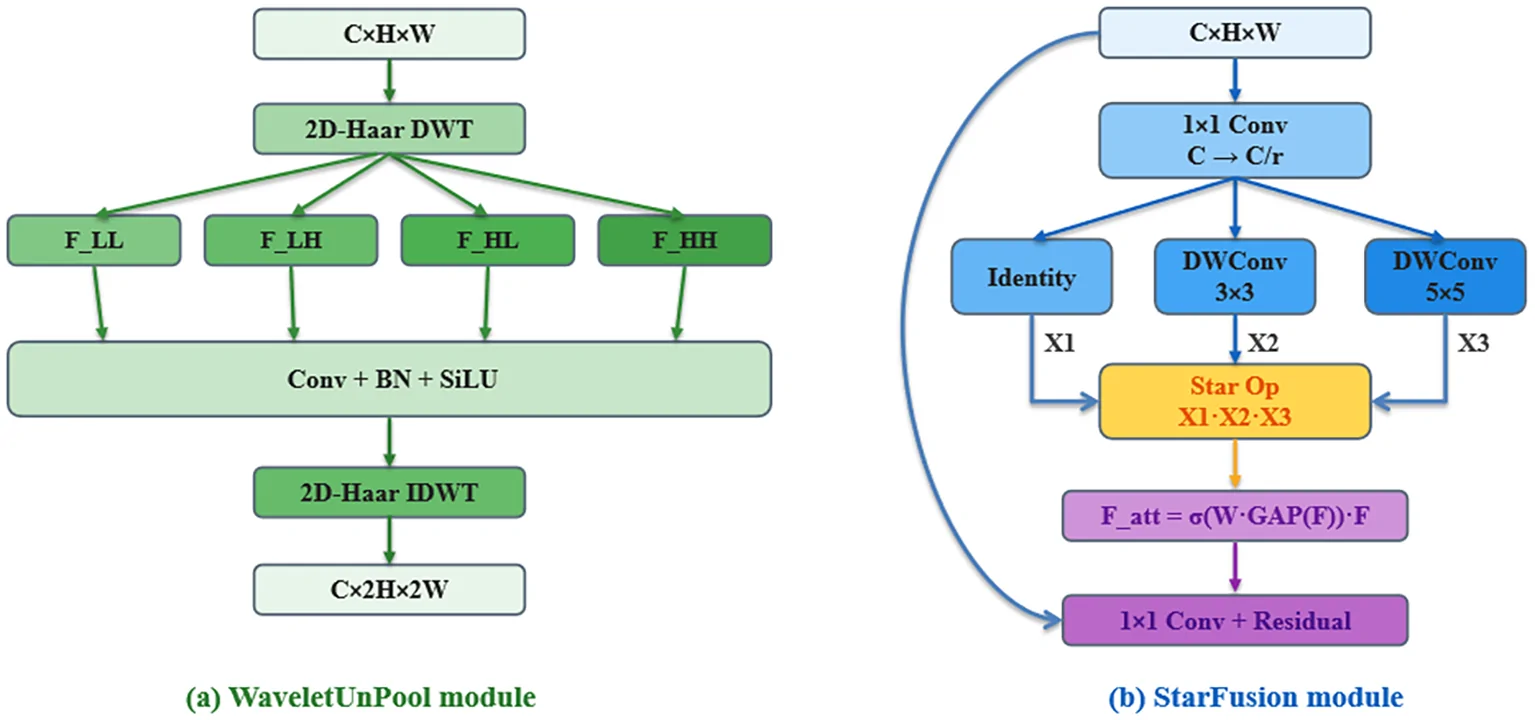
Structure of the WSF (Wavelet Unpooling StarFusion) network. **(a)** the WaveletUnPool wavelet-based upsampling module; **(b)** the StarFusion feature enhancement module.

#### 2.2.3 NWD loss function optimization

The bounding-box regression loss of YOLOv11n adopts CIoU (Complete Intersection over Union) (Zheng et al., 2022) (which introduces an aspect-ratio consistency constraint on top of DIoU (Zheng et al., 2020)), defined in Equation 5:

(5) $$\text{L\_CIoU}=1-\text{IoU}+{\text{ρ}}^{2}\left(\text{b, b\_gt}\right)/{\text{c}}^{2}+\alpha \text{v}$$

where IoU is the intersection over union between the predicted and ground-truth boxes, ρ^2^(b, b_gt) is the squared Euclidean distance between the centers of the two boxes, c is the diagonal length of the smallest enclosing rectangle, and αv is the aspect-ratio consistency penalty term.

CIoU performs well for objects of conventional size, but has the following shortcomings for extremely small objects such as thrips (Wang et al., 2021; Zheng et al., 2022): (1) On a 640×640 feature map, a 10-pixel thrips occupies only about 2×2 feature points, and IoU is extremely sensitive to position offset—as shown in **Figure 3a**, based on the typical 13 px, 23.5 px, and 34.5 px objects (corresponding to small, medium, and large scale quantiles, respectively) in our thrips dataset, at a 2-pixel offset the IoU drops from 1.0 to about 0.56, 0.72, and 0.80; the smaller the object, the more sharply IoU decreases, i.e., the weaker the effective gradient CIoU provides for extremely small objects. In other words, the smaller the object, the more sensitive IoU is to offset and the sharper its decline, leaving CIoU unable to provide effective gradient guidance; (2) the various metrics of CIoU (center distance, aspect ratio, etc.) have low signal-to-noise ratio on extremely small objects, which may mislead the model’s optimization direction; and (3) in dense scenarios, CIoU can hardly distinguish adjacent thrips individuals, leading to missed or duplicated detections. This “the smaller the object, the more sensitive IoU” phenomenon has been systematically verified by Wang et al. (2021) on the AI-TOD dataset, and we observe a consistent pattern on the thrips dataset (**Figure 3a**).

**Figure 3:**
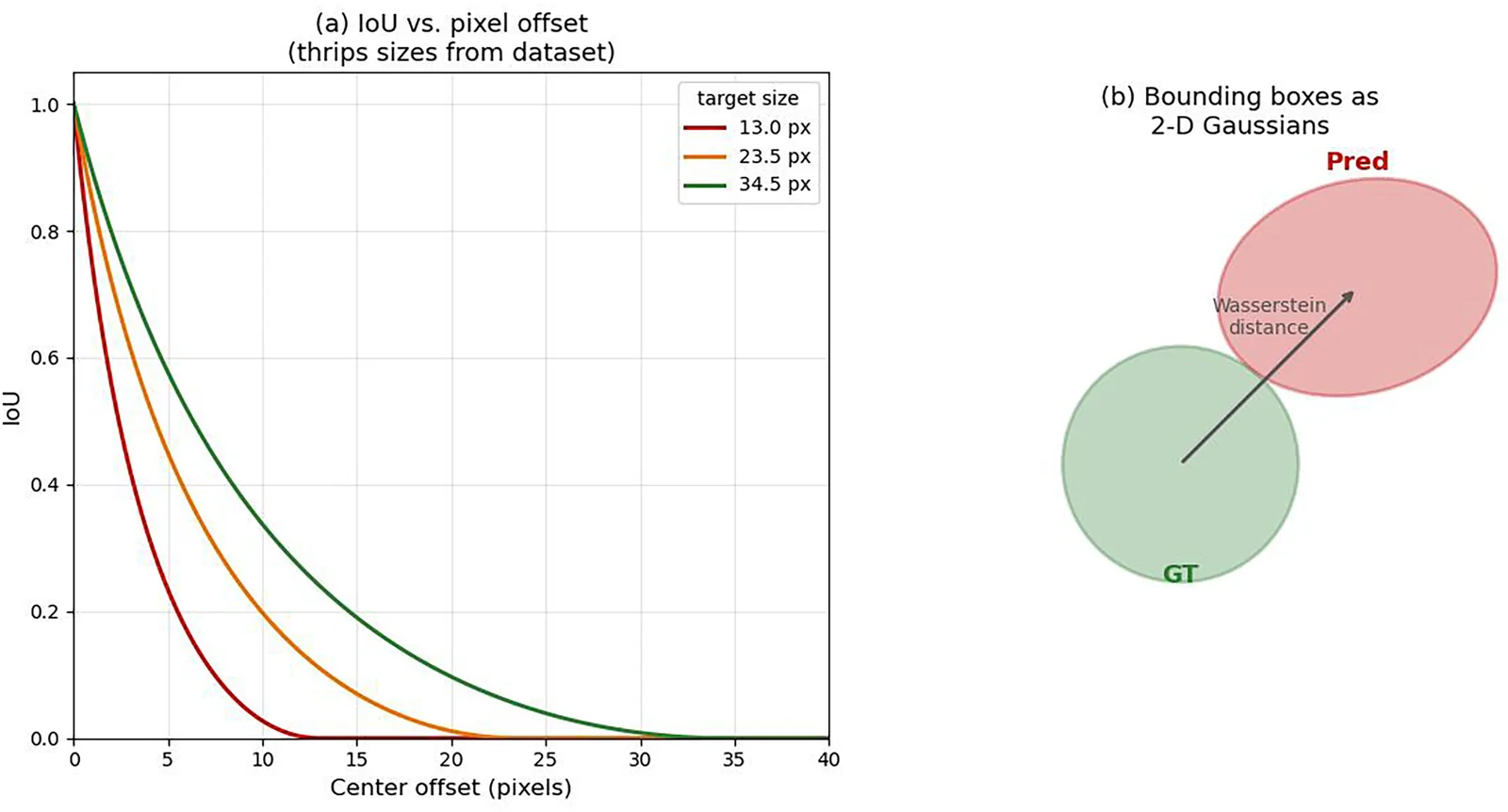
Principle analysis of the NWD loss function (**(a)** is plotted from the annotation-box statistics of our thrips dataset, D = 23 px); panel (b) illustrates the ground-truth (GT) and predicted boxes modeled as two-dimensional Gaussian distributions, between which the Wasserstein distance is computed.

To solve the above problems, this paper introduces the Normalized Wasserstein Distance (NWD) as an auxiliary regression loss (Wang et al., 2021). NWD models the object bounding box as a two-dimensional Gaussian distribution (as shown in **Figure 3b**, the predicted and ground-truth boxes correspond to an elliptical Gaussian distribution each, and the Wasserstein distance between the two distributions is a comprehensive measure of position and shape difference), and measures the difference between the two distributions through the Wasserstein distance, providing a smooth and continuous gradient response to small-object position offsets. As shown in **Figure 3a**, under the same position offset, NWD changes gently across the entire offset range, whereas IoU (especially for small objects) plummets to near zero at a tiny offset; therefore NWD can provide continuous and effective gradient guidance to complement CIoU (Wang et al., 2021).

Specifically, for a bounding box R = (cx, cy, w, h), where (cx, cy) are the center coordinates and (w, h) are the width and height, it is modeled as a two-dimensional Gaussian distribution N(μ, Σ), as shown in Equation 6:

(6) μ=[cx, cy]^T, ∑=[w2/4, 0; 0, h2/4]

i.e., the center of the elliptical Gaussian distribution lies at the bounding-box center, and the covariance matrix is proportional to the bounding-box size.

The second-order Wasserstein distance between the two-dimensional Gaussian distributions μ_1_ = N(m_1_, Σ_1_) and μ_2_ = N(m_2_, Σ_2_) corresponding to two bounding boxes is defined in Equation 7:

(7) W22(μ1,μ2)=‖m1−m2‖22+‖∑1^{1/2}−∑2^{1/2}‖_F2

where ||·||_2_ is the Euclidean norm and ||·||_F is the Frobenius norm. The first term measures the center-position difference and the second term measures the shape and scale difference.

Further normalizing the Wasserstein distance to the interval, as shown in Equation 8:

(8) $$\text{NWD}\left(\text{N\_a, N\_b}\right)=\exp\left(-\frac{\sqrt{({\text{W}}_{2}^{2}\left(\text{N\_a, N\_b})\right)}}{\text{D}}\right)$$

where D is the normalization constant, empirically set to D = 23 (pixels) in this paper based on the statistics of object scales in the dataset. Specifically, we computed the average side length s = (w + h)/2 (w, h in pixels) of all thrips annotation boxes in the dataset and obtained an average object scale <s> ≈ 23.05 px and a median of 22.5 px. Setting D to the dataset’s average object scale makes NWD ≈ exp(−1) ≈ 0.37 when the two box centers are offset by about one object scale, while at an offset of only 1–2 px NWD decreases only slightly (≈ 0.96, 0.92)—that is, using the object’s own scale as the unit gives a smooth, mild response to the pixel-level localization error of extremely small objects, providing NWD with a scale benchmark consistent with the characteristics of tiny thrips. The NWD loss function is defined in Equation 9:

(9) L_NWD=1−NWD(N_a, N_b)

NWD responds smoothly to small-object position deviation: when the two boxes are completely non-overlapping, IoU = 0 causes gradient vanishing, whereas NWD can still provide an effective gradient signal; when the two boxes deviate by only 1–2 pixels, IoU changes abruptly while NWD changes gently, enabling more stable guidance of model optimization. Specifically, the overlap term of IoU has a constant zero gradient when the two boxes do not overlap, so that extremely small objects cannot obtain an effective localization gradient in the early training stage because the predicted and ground-truth boxes are almost non-overlapping; NWD transforms the position deviation into a continuous distribution distance through Gaussian-distribution modeling and maintains a non-zero, smooth gradient response throughout the offset range, thereby effectively alleviating the localization-signal vanishing problem of small objects. In addition, NWD naturally captures the shape and scale information of bounding boxes through distribution modeling, and can more effectively distinguish adjacent objects in dense scenarios.

Notably, simply replacing an IoU-type loss with NWD, although it can alleviate small-object missed detection, may affect convergence stability due to the lack of direct constraints on the geometric shape of bounding boxes. Therefore, several subsequent studies adopt a joint strategy of NWD and an IoU-type loss to combine the advantages of both: Chen et al. (2025) proposed the NWD-CIoU loss for small remote-sensing object detection, which reduces the small-object missed-detection rate while retaining CIoU’s regression ability for large objects; Sun et al. (2025) constructed a CIoU-NWD hybrid loss to alleviate the prediction-deviation problem of dense small objects from a UAV perspective.

Inspired by such joint-loss strategies, this paper adopts a weighted combination of CIoU and NWD as the bounding-box regression loss, as shown in Equation 10:

(10) L_reg=λ_CIoU·L_CIoU+λ_NWD·L_NWD

where λ_CIoU and λ_NWD are weighting coefficients, set to 0.5 and 0.5 respectively in this paper; experiments verified the effectiveness of this combination.

#### 2.2.4 Lightweight EfficientHead detection head

The decoupled detection head of YOLOv11n performs classification and regression predictions on three feature layers of different scales (80×80, 40×40, 20×20). Each detection-head branch contains two 3×3 convolutional layers and one 1×1 output convolutional layer, where the standard 3×3 convolution (groups = 1) aggregates the features of all input channels for every output channel along the channel dimension, i.e., there is a dense connection between input and output channels. For high-dimensional feature channels, this cross-channel dense connection brings a large amount of parameters and computational overhead (Ioannou et al., 2017).

To reduce the parameters and computation of the detection head, this paper designs the lightweight EfficientHead detection head, whose core improvement is to replace the standard 3×3 convolution with a grouped convolution (GroupConv). Grouped convolution evenly divides the input channels into G groups, each performing convolution independently without inter-group channel interaction. This reduces the convolution-kernel parameter count to 1/G of the original, and the computation is reduced accordingly.

Specifically, for a convolutional layer with input channels C_in and output channels C_out:


$$\text{Standard convolution parameters: Params\_std}=\text{C\_in}\times {\text{K}}^{2}\times \text{C\_out}$$

$$\text{Grouped convolution parameters: Params\_group}=\left(\text{C\_in}/\text{G}\right)\times {\text{K}}^{2}\times \left(\text{C\_out}/\text{G}\right)\times G=\text{C\_in}\times {\text{K}}^{2}\times \text{C\_out}/\text{G}$$

That is, the parameter count of grouped convolution is 1/G of that of standard convolution. EfficientHead uses G = 8, reducing the parameters and computation of the 3×3 convolutional layer by about 87.5%.

The structure of each detection branch of EfficientHead is: GroupConv 3×3 (G = 8) → BatchNorm → SiLU → GroupConv 3×3 (G = 8) → BatchNorm → SiLU → Conv 1×1 (classification/regression output). SiLU activation and batch normalization (Batch Normalization) between the grouped convolutions provide non-linear transformation and training stability. Although grouped convolution loses inter-group channel interaction, partial cross-group information fusion is achieved through the subsequent point-wise 1×1 convolution, and the integrity of feature propagation is ensured by a residual skip connection. The structure comparison of EfficientHead is shown in **Figure 4**.

**Figure 4:**
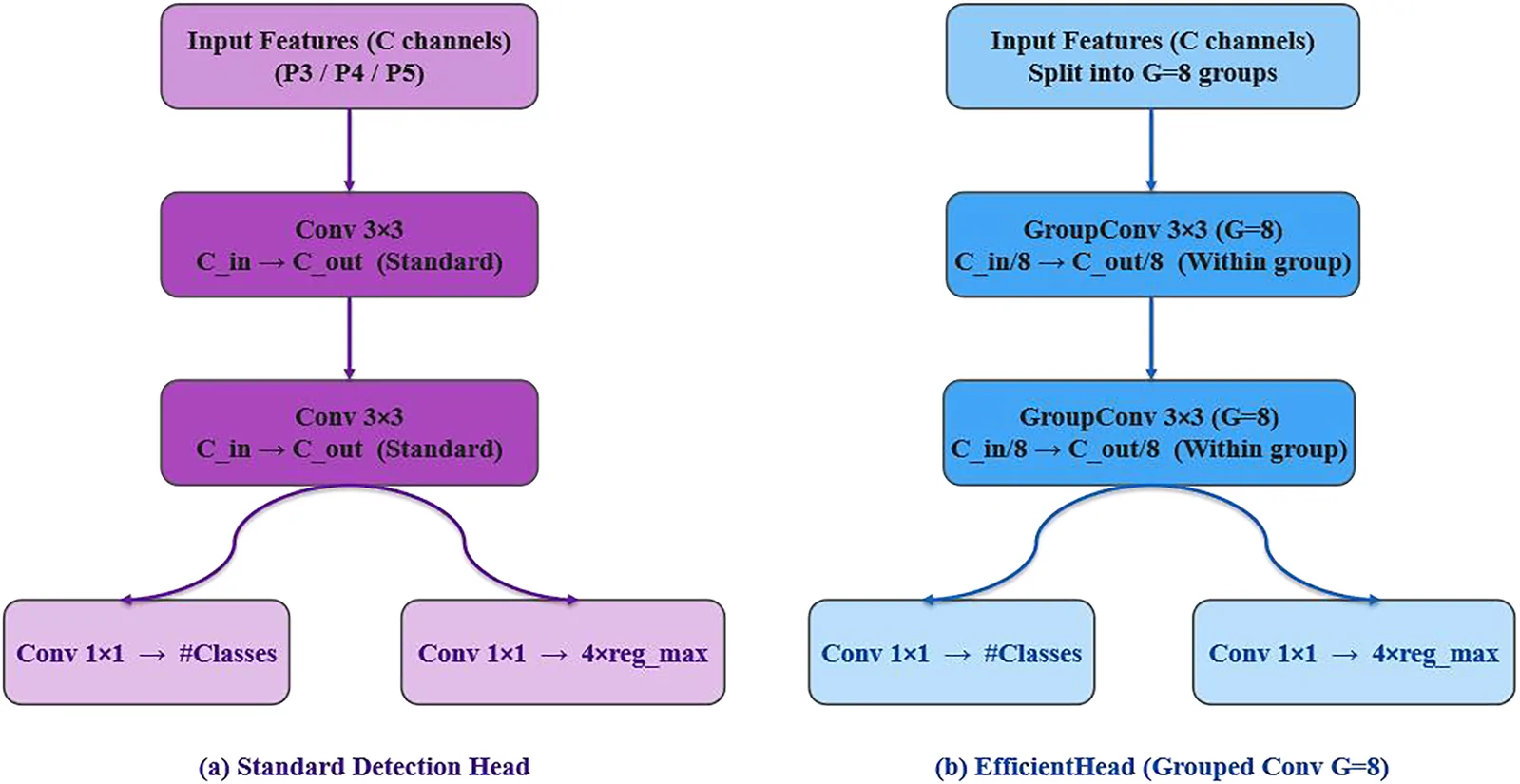
Structure comparison of the lightweight EfficientHead detection head.

### 2.3 Model evaluation metrics

This paper adopts the commonly used evaluation metrics in object detection to quantitatively assess model performance, including precision (P), recall (R), mean average precision (mAP), and model lightweighting and inference-speed metrics.

The definitions of precision and recall are in Equation 11:

(11) P=TP/(TP+FP), R=TP/(TP+FN)

where TP is a true positive (thrips correctly detected), FP is a false positive (background misdetected as thrips), and FN is a false negative (thrips missed). The IoU threshold is set to 0.5, i.e., a detection is considered correct when the IoU between the predicted and ground-truth boxes is ≥ 0.5.

The mAP@0.5 is the mean of the average precisions of all classes at the IoU = 0.5 threshold, calculated using Equation 12:

(12) mAP@0.5=(1/N)∑_{c=1}^{N}AP_c@0.5

Since this paper detects only a single class of thrips (N = 1), mAP@0.5 is numerically equivalent to the average precision AP of this single class; hence “mAP” hereinafter refers to this single-class precision. In addition, mAP@0.5:0.95 (the mean of mAP at IoU thresholds from 0.5 to 0.95 with a step of 0.05) is adopted as a stricter evaluation metric.

The model lightweighting evaluation metrics include: model parameter count (Params, M), floating-point operations (GFLOPs), and model file size (MB, fp32 precision). In addition, inference speed is measured in frames per second (FPS). FPS is benchmarked with a batch size of 1 at an input size of 640×640: 50 warm-up inferences are executed first, and the reported value is the average of the subsequent 200 timed inferences. Both GPU inference (NVIDIA GeForce RTX 4090D) and CPU inference (Intel i7-11700) are reported, since edge deployment may run on either platform.

## 3 Results

### 3.1 Experimental environment and parameter settings

The training, validation, and testing experiments in this study were conducted under the same hardware and software environment. Hardware configuration: CPU Intel i7-11700 @ 2.5 GHz, 32 GB memory, GPU NVIDIA GeForce RTX 4090D with 24 GB memory. Software environment: operating system Ubuntu 22.04 LTS, deep-learning framework PyTorch 2.0, CUDA version 11.8, programming language Python 3.9.

The training hyperparameters were set as follows: input image size 640×640 pixels, batch size 64, initial learning rate 0.01 with a cosine-annealing learning-rate decay strategy, optimizer SGD (momentum 0.937, weight decay 5×10^−4^), and training epochs 300. A warm-up strategy was adopted in the first 3 epochs, with the learning rate linearly increasing from 0.001 to 0.01.

### 3.2 WSF module performance evaluation

To verify the effectiveness of the WaveletUnPool and StarFusion components in the WSF neck network, comparison experiments were conducted on the YOLOv11n baseline by separately replacing the upsampling module and the feature-fusion module in the neck, on the thrips dataset. All experiments kept the other conditions and hyperparameters unchanged.

As can be seen from **Table 3**, compared with nearest-neighbor interpolation, WaveletUnPool upsampling improves mAP@0.5 by 1.2 percentage points (87.4% → 88.6%), indicating that wavelet-based upsampling (WaveletUnPool) improves the recovery quality of small-object features by preserving high-frequency detail. After introducing StarFusion star-shaped fusion, mAP@0.5 is further improved to 91.2%, an increase of 2.6 percentage points, verifying the effectiveness of the star operation in capturing high-order interactions of multi-scale features. Moreover, compared with transposed convolution (TransConv), WaveletUnPool reduces parameters by 0.27M (2.71M vs. 2.98M) yet achieves a higher mAP (91.2% vs. 89.7%), demonstrating the dual advantage of wavelet transform in both accuracy and efficiency.

**Table 3:** Performance comparison of WSF module components.

Upsampling method	Feature fusion	P (%)	R (%)	mAP@0.5 (%)	Params (M)	GFLOPs
Nearest (baseline)	Concat	83.7	79.9	87.4	2.58	6.3
WaveletUnPool	Concat	85.1	81.3	88.6	2.58	6.5
Bilinear	StarFusion	86.2	82.5	89.4	2.71	6.8
WaveletUnPool	StarFusion	87.8	84.1	91.2	2.71	7.0
TransConv	StarFusion	86.5	82.8	89.7	2.98	7.5

### 3.3 NWD loss function comparison experiments

To verify the effectiveness of the NWD loss in thrips small-object detection, the baseline YOLOv11n model was trained with different regression loss functions while keeping other parameters and the structure unchanged.

As can be seen from **Table 4**, using the NWD loss alone improves mAP@0.5 from the CIoU baseline of 87.4% to 90.1%, an increase of 2.7 percentage points, and improves recall by 3.6 percentage points (79.9% → 83.5%), indicating that NWD is particularly effective in reducing small-object missed detection. When CIoU and NWD are combined, mAP@0.5 reaches 91.0%, and precision, recall, and mAP are all optimal, verifying the complementarity of the two loss functions: CIoU maintains the box-shape consistency constraint while NWD provides a smooth position gradient.

**Table 4:** Comparison of different bounding-box regression loss functions.

Loss function	P (%)	R (%)	mAP@0.5 (%)	mAP@0.5:0.95 (%)
CIoU (baseline)	83.7	79.9	87.4	55.6
GIoU	83.1	78.5	86.8	54.2
DIoU	83.9	80.2	87.7	55.9
WIoU	85.2	82.3	89.4	57.6
NWD	86.8	83.5	90.1	58.9
CIoU + NWD	87.6	84.2	91.0	59.8

### 3.4 EfficientHead lightweight comparison experiments

To verify the effect of the EfficientHead grouped detection head on model compression, EfficientHeads with different group numbers G were used to replace the YOLOv11n baseline detection head in comparison experiments.

As can be seen from **Table 5**, as the group number G increases, the model parameters and computation continue to decrease. At G = 8, mAP@0.5 decreases by only 0.3 percentage points (87.4% → 87.1%), but the parameter count decreases by 23.3% (2.58M → 1.98M), GFLOPs decreases by 15.9% (6.3 → 5.3), and the model size is reduced to 4.2 MB, achieving a good balance between accuracy and efficiency. At G = 16 the accuracy drops markedly (by 1.6 percentage points), indicating that excessive grouping impairs the feature-interaction ability of the detection head. Therefore, the G = 8 EfficientHead configuration is adopted in the subsequent experiments.

**Table 5:** Performance comparison of EfficientHead with different group numbers G.

Group number G	P (%)	R (%)	mAP@0.5 (%)	Params (M)	GFLOPs	Model size (MB)
Standard head (baseline)	83.7	79.9	87.4	2.58	6.3	5.5
G=2	83.8	80.1	87.5	2.31	5.8	4.9
G=4	83.5	79.8	87.3	2.12	5.5	4.5
G=8	83.4	79.6	87.1	1.98	5.3	4.2
G=16	82.1	78.3	85.8	1.89	5.1	4.0

### 3.5 Ablation study

To systematically verify the independent contribution and complementary effect of each improved module on model performance, a comprehensive ablation study was conducted on the YOLOv11n baseline, gradually introducing the three improved modules WSF, NWD loss, and EfficientHead.

As can be seen from **Table 6**, the effects of the three improved modules on model performance are as follows:

**Table 6:** Ablation study results.

No.	WSF	NWD	EfficientHead	P (%)	R (%)	mAP@0.5 (%)	Params (M)	GFLOPs
1	—	—	—	83.7	79.9	87.4	2.58	6.3
2	✓	—	—	87.8	84.1	91.2	2.71	7.0
3	—	✓	—	86.8	83.5	90.1	2.58	6.3
4	—	—	✓	83.4	79.6	87.1	1.98	5.3
5	✓	✓	—	88.5	85.3	91.8	2.71	7.0
6	✓	✓	✓ (ours)	88.2	85.0	91.5	2.11	6.0

1. When the WSF module is introduced alone (Experiment 2), mAP@0.5 improves from 87.4% to 91.2%, an increase of 3.8 percentage points, and recall improves from 79.9% to 84.1%, an increase of 4.2 percentage points. This shows that WSF, through wavelet upsampling to preserve spatial details and star-shaped fusion to enhance feature interaction, improves the detection and localization ability for small thrips objects. However, WSF introduces a small amount of extra computation (GFLOPs increases from 6.3 to 7.0).
2. When the NWD loss is introduced alone (Experiment 3), mAP@0.5 improves to 90.1% and precision improves by 3.1 percentage points (83.7% → 86.8%). Without increasing parameters or computation, NWD alone improves small-object localization accuracy by optimizing the loss function.
3. When EfficientHead is introduced alone (Experiment 4), the parameter count drops from 2.58M to 1.98M (down 23.3%), GFLOPs drops from 6.3 to 5.3 (down 15.9%), and mAP@0.5 drops by only 0.3 percentage points, proving the effectiveness of the grouped-convolution detection head in model compression.
4. When the three modules are combined (Experiment 6), the overall mAP@0.5 is 91.5%, an improvement of 4.1 percentage points over the baseline; the parameter count drops to 2.11M (down 18.2%); and GFLOPs drops to 6.0 (down 4.8%). Compared with the WSF + NWD combination (Experiment 5), EfficientHead reduces the parameter count by 22.1% (2.71M → 2.11M) at the cost of only 0.3 percentage points of mAP, achieving the optimal balance between accuracy and efficiency.

In summary, the proposed YOLOv11n-WNE model achieves the best overall performance in both detection accuracy and model lightweighting, verifying that the three improvements act on complementary stages of the detection pipeline—feature representation (WSF), localization supervision (NWD), and computational efficiency (EfficientHead)—and that their combination achieves the optimal accuracy–efficiency balance.

### 3.6 Comparison with mainstream detection models

To further verify the comprehensive performance of our model, YOLOv11n-WNE was compared with current mainstream object-detection models, including YOLOv5s (Jocher et al., 2023), YOLOv7-tiny (Wang et al., 2023), YOLOv8n (Jocher et al., 2023), YOLOv11n (Jocher et al., 2024), and RT-DETR (Zhao et al., 2024). To justify the choice of the baseline, the adjacent releases YOLOv10n (Wang et al., 2024) and YOLOv12n (Tian et al., 2025) were additionally trained and tested under identical settings. Moreover, to position the proposed method within the tiny-object detection literature, two schemes specifically designed for small objects were also compared: a TPH-style transformer prediction head built on YOLOv5n (Zhu et al., 2021) and a BiFPN-neck variant of YOLOv11n (Tan et al., 2020). Since an NWD-augmented variant of the YOLOv11n baseline is already evaluated in **Table 6** (Experiment 3), it serves as the direct reference for NWD-based comparison. All models were trained, validated, and tested under the same dataset and experimental environment.

As can be seen from **Table 2**, our YOLOv11n-WNE model shows significant advantages in both accuracy and lightweighting:

1. Detection accuracy: mAP@0.5 reaches 91.5%, which is 20.8, 4.5, 24.7, 5.3, and 4.1 percentage points higher than RT-DETR, YOLOv5s, YOLOv7-tiny, YOLOv8n, and YOLOv11n, respectively. The stricter mAP@0.5:0.95 metric reaches 60.2%, also ranking best among all compared models.
2. Lightweighting: the parameter count is only 2.11M, which is 81.8% of YOLOv11n (2.58M), 30.1% of YOLOv5s (7.02M), and 6.6% of RT-DETR (32.0M). The model size is only 4.5 MB, the smallest among all models. GFLOPs is 6.0, lower than YOLOv11n (6.3), YOLOv8n (8.1), and YOLOv5s (15.8).
3. Inference speed: measured under the protocol described in Section 2.3, YOLOv11n-WNE achieves 123.8 FPS on the GPU and 14.5 FPS on the CPU, consistent with its low computational cost (6.0 GFLOPs) and small model size (4.5 MB) and confirming its suitability for real-time edge deployment. The per-model speed comparison is given in **Table 2**.
4. Overall trade-off: our model achieves the highest detection accuracy with the smallest parameter count and model size, demonstrating that the three complementary improvement strategies—WSF feature enhancement, NWD loss optimization, and the EfficientHead lightweight detection head—jointly deliver this balance: WSF and NWD improve detection accuracy through feature representation and localization supervision, respectively, while EfficientHead reduces model complexity at a negligible accuracy cost. The visualization of the performance comparison among the mainstream models is shown in **Figure 5**.

**Figure 5:**
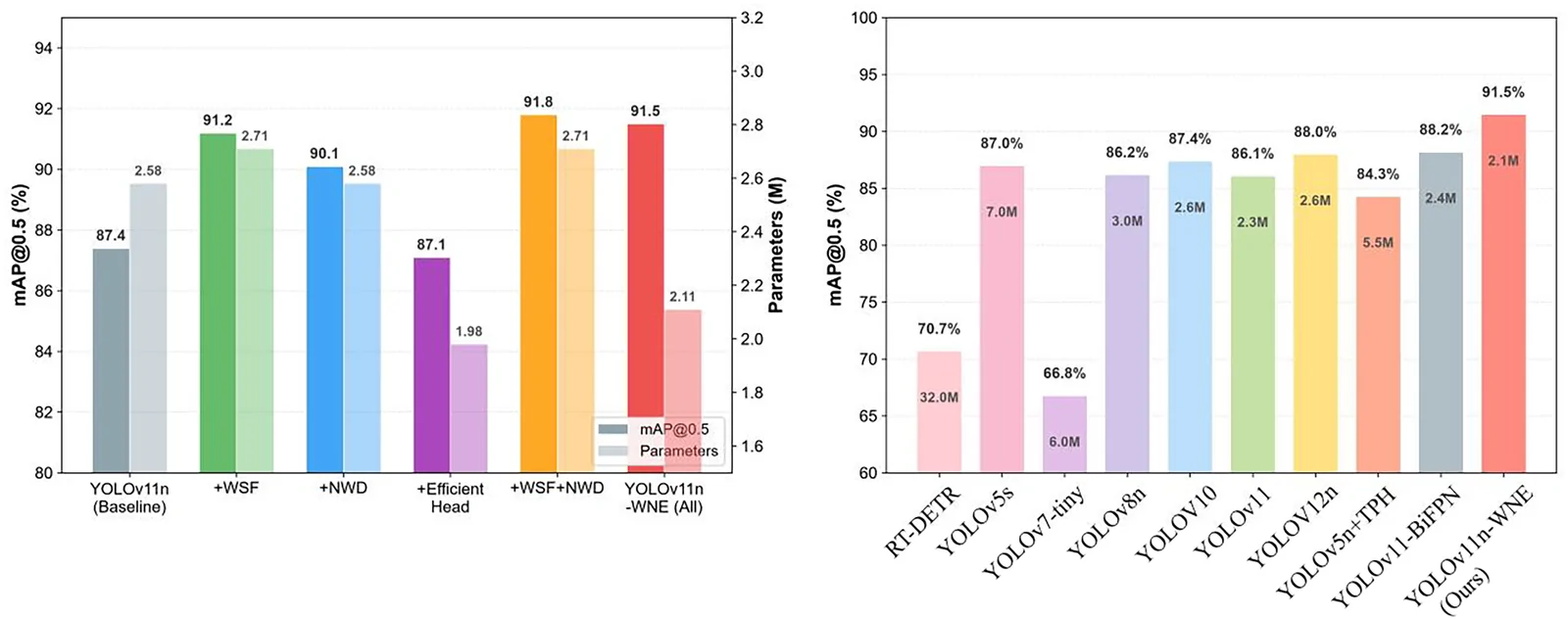
Performance comparison of the models. **(a)** Ablation Study Results; **(b)** Model Comparison.

### 3.7 Visualization analysis of detection results

To intuitively show the detection effect of the improved model, the detection results of the YOLOv11n baseline and the improved YOLOv11n-WNE were visually compared under three typical thrips distribution scenarios: low-density, medium-density, and high-density.

In the low-density scenario (1–5 thrips per image), the baseline YOLOv11n model can already detect most thrips objects well, but occasionally misdetects debris with similar texture as thrips. Through the WSF network, the YOLOv11n-WNE model enhances feature discriminability and suppresses background-noise interference, so that false detections basically disappear.

In the medium-density scenario (6–20 thrips per image), the baseline model shows a small number of missed detections, especially when thrips have similar color to the sticky-trap background or are located in edge regions, where the missed-detection rate increases. Through the smooth position gradient of the NWD loss, the improved model maintains a high detection rate in boundary regions and low-contrast regions, and recall improves from 79.9% to 85.0%.

In the high-density scenario (more than 20 thrips per image)—the scenario that best demonstrates the advantage of the improved model—the baseline model suffers serious missed detections, especially in regions where thrips cluster and overlap, where it tends to merge multiple adjacent small individuals into one detection or simply miss them. Benefiting from the spatial details preserved by the WSF network and the discrimination ability of NWD for dense distributions, the improved model can still accurately detect a large number of thrips individuals in highly overlapping dense regions, reducing the missed-detection and false-merging rates, and yielding more complete and accurate detection results. The visualization comparison of detection results under different density scenarios is shown in **Figure 6**.

**Figure 6:**
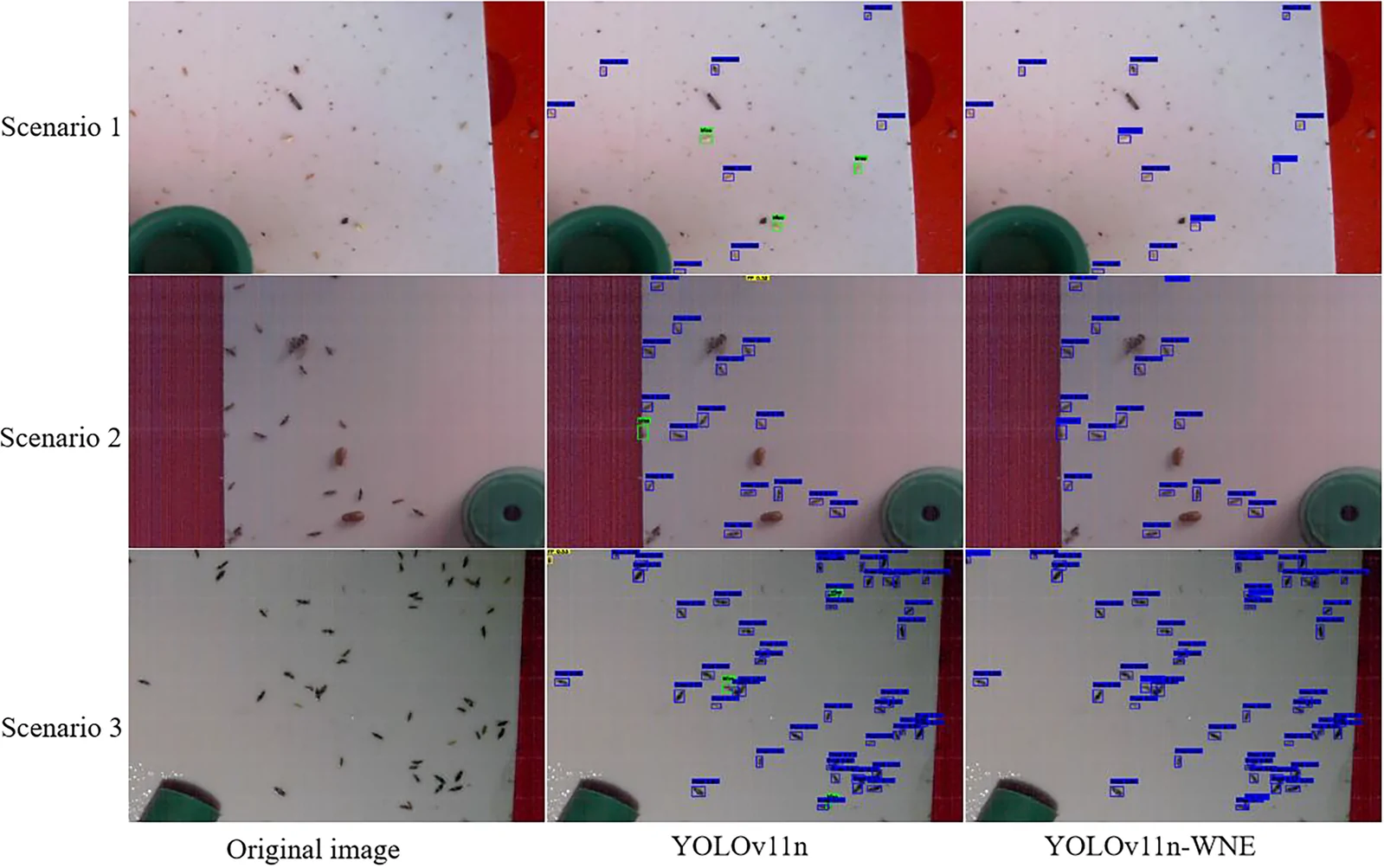
Visualization of thrips detection results.

### 3.8 Quantitative analysis of false positives

Sticky traps deployed in greenhouses inevitably accumulate dust particles, non-target insects, and plant debris, which are the main potential sources of false positives. To quantify the model’s robustness against these distractors, all false positives produced by the baseline YOLOv11n and the improved YOLOv11n-WNE on the 360 test images were extracted at the same low confidence threshold and manually categorized by their visual content. This count-based protocol differs from the mAP@0.5 evaluation used in **Table 2**, which sweeps the confidence score with the IoU threshold fixed at 0.5. As summarized in **Table 7**, the improved model reduces the total number of false positives from 720 to 360 (i.e., false positives per image from 2.0 to 1.0, a 50.0% reduction), and the remaining false positives are dominated by non-target insects (138/360 = 38.3%), which substantially overlap with thrips in size and texture. These results indicate that the feature discriminability enhanced by the WSF network suppresses most texture-level confusions—especially dust particles (reduced from 30.6% to 21.7% of all FPs) and background textures (reduced from 26.4% to 20.0%)—while the residual errors mainly originate from non-target insects that share the 10–40 pixel size range of thrips, which we consider the most promising target for further improvement through distractor-aware negative-sample augmentation.

**Table 7:** Categorization of false positives on the test set.

FP category	YOLOv11n (count/share)	YOLOv11n-WNE (count/share)
Dust and impurity particles	220 (30.6%)	78 (21.7%)
Non-target insects	165 (22.9%)	138 (38.3%)
Plant debris	145 (20.1%)	72 (20.0%)
Background texture and other	190 (26.4%)	72 (20.0%)
Total (FPPI)	720 (2.0 FPPI)	360 (1.0 FPPI)

## 4 Discussion

### 4.1 Mechanism of the WSF network in improving small-object detection

In traditional neck networks, information loss during upsampling is one of the key factors limiting small-object detection accuracy. The WaveletUnPool proposed in this paper decomposes features into low-frequency and high-frequency components through the Haar wavelet transform, preserving edge and texture details during upsampling reconstruction and providing richer spatial localization information for the subsequent detection head. The ablation results (**Table 6**) show that introducing the WSF module alone improves recall from 79.9% to 84.1%, an increase of 4.2 percentage points, demonstrating the importance of spatial-detail preservation for small-object detection.

The StarFusion star-shaped fusion module implicitly establishes a multi-dimensional non-linear relationship in the feature space through element-wise multiplicative interaction. Compared with traditional addition/concatenation fusion, multiplicative interaction can expand the expressive ability of features in an exponential space (Ma et al., 2024), enabling richer feature representation under a limited computation budget. This design is particularly effective in high-density small-object scenarios such as thrips detection—individuals in dense regions exhibit complex visual co-occurrence patterns, and multiplicative interaction can better capture these patterns.

### 4.2 Complementary mechanism between NWD and CIoU

In extremely small-object detection, the gradient response of IoU-based loss functions (including CIoU) exhibits a non-linear abrupt-change characteristic when the object scale is small: when the predicted box deviates by only 1–2 pixels, the IoU value may plummet from a high value to near zero, causing gradient instability. NWD transforms the position deviation into a continuous distance in the distribution-measurement space through Gaussian-distribution modeling, providing a smooth gradient response (Wang et al., 2021). The experimental results (**Table 4**) show that using NWD alone improves recall from 79.9% to 83.5%, confirming the prominent effect of NWD in alleviating small-object missed detection.

However, the pure NWD loss lacks direct constraints on the bounding-box shape, and several studies (Chen et al., 2025; Sun et al., 2025) point out that simply replacing it may slow down convergence. Therefore, this paper adopts a weighted combination strategy of CIoU and NWD, which is consistent with the NWD-CIoU joint-loss design of Chen et al. (2025) and Sun et al. (2025)—CIoU maintains box-shape consistency while NWD provides a smooth position gradient, forming a complementarity. The ablation experiment (**Table 6**, Experiment 5) confirms the effectiveness of this complementary design.

### 4.3 Accuracy–efficiency trade-off of EfficientHead

Grouped convolution reduces parameters and computation by dividing channels into independent groups, but inevitably loses inter-group channel interaction. The G = 8 configuration of EfficientHead achieves 23.3% parameter compression and 15.9% computation reduction (**Table 5**) at the cost of a minimal accuracy loss (mAP@0.5 drops by only 0.3 percentage points), indicating a high degree of redundancy among the channels of the detection head. The subsequent 1×1 convolution and cross-layer residual connection partially compensate for the inter-group information loss, which is also the key design enabling EfficientHead to basically maintain accuracy while compressing.

### 4.4 Comparison with related work

This work shares certain architectural components with two recent studies that also target sticky-trap or agricultural pest detection using improved YOLOv11n, and it is important to clarify the differentiated contributions.

Zeng et al. (2025) proposed a lightweight tea pest and disease recognition method based on improved YOLOv11n, employing a WSF (WaveletUnPool–StarFusion) network and an EfficientHead with grouped convolutions. The present study adopts the same WSF network and EfficientHead architecture; however, the key differences are threefold. First, the application domain differs fundamentally: Zeng et al. target tea diseases whose lesion areas are typically hundreds of pixels, whereas this study addresses thrips on sticky traps that occupy only 10–40 pixels—a far more challenging tiny-object scenario. Second, Zeng et al. do not incorporate any specialized small-object regression loss; in contrast, this study introduces a NWD–CIoU joint loss whose normalization constant D is calibrated to the thrips object scale (<s> ≈ 23 px), providing smooth gradient guidance that is critical for pixel-level localization of extremely small objects. Third, this study constructs a dedicated thrips dataset (2, 400 images, 40, 480 instances) with image-slice preprocessing specifically designed to increase the pixel proportion of tiny pests. Therefore, the principal novelty of this work lies not in the individual WSF or EfficientHead modules, but in the problem-driven integration of these components with the NWD joint loss strategy and the purpose-built thrips dataset for the sticky-trap tiny-object detection scenario. The ablation results (**Table 6**) further indicate that WSF and NWD alleviate complementary failure modes—feature degradation of tiny objects and unstable localization gradients, respectively—while EfficientHead contributes efficiency rather than accuracy; their combination is therefore best characterized as a complementary, accuracy–efficiency-oriented design rather than an additive accumulation of individual gains.

Meng et al. (2026) proposed a sticky-trap pest detection method based on improved YOLOv11n (YOLOv11n-LMN), achieving mAP@0.5 of 91.3% by introducing a PP-LCNet lightweight backbone, a MulSimAM attention mechanism, and the NWD loss, combined with SAHI slice-aided inference. Although both studies address sticky-trap pest detection and employ NWD, the methodological strategies differ significantly. Methodologically, Meng et al. replace the backbone with PP-LCNet and enhance features with MulSimAM attention, whereas this study retains the original YOLOv11n backbone and instead improves the neck with the WSF network (wavelet upsampling + star-shaped fusion) and compresses the detection head with EfficientHead. At inference time, Meng et al. rely on SAHI slice-aided inference to improve small-object detection, which increases inference latency; this study achieves comparable accuracy (91.5% vs. 91.3%) without SAHI, through architectural improvements alone, yielding a lighter and faster inference pipeline. Furthermore, Meng et al. detect multiple pest species (thrips and whiteflies), whereas this study focuses on single-class thrips detection with a larger dedicated dataset (2, 400 images vs. their reported scale), enabling more detailed analysis of the tiny-object detection challenges specific to thrips. These differences demonstrate that the two approaches are complementary rather than redundant, and the WSF + NWD + EfficientHead combination offers a distinct and competitive alternative for sticky-trap thrips monitoring.

A closely related line of work targets microscopic nematode detection rather than thrips. Erdinç and Erdoğan (2026) proposed LightDetectorMS, an ultra-lightweight anchor-free detector for entomopathogenic nematodes in laboratory suspensions, reporting mAP@0.5 of 0.9119 with only 62, 991 parameters (0.46 MB) and 152.5 FPS under five-fold cross-validation (coefficient of variation<5%). Although the imaging modality differs (three-dimensional dense suspension vs. two-dimensional sticky-trap surface) and the target scale in their setting is roughly an order of magnitude smaller, the methodological parallels are notable: YOLOv5s serves as a common reference baseline in both studies; Erdinç and Erdoğan (2026) additionally use YOLOv12s, while our study uses a broader set including YOLOv7-tiny, YOLOv8n, YOLOv10n, YOLOv11n, and YOLOv12n and adopt anchor-free, lightweight backbones to balance accuracy with deployment cost. The principal difference in reporting practice is that Erdinç and Erdoğan (2026) use five-fold cross-validation and report the coefficient of variation across folds, whereas we report a single training run on a held-out test split. This methodological gap, rather than any algorithmic incompatibility, is the primary reason our results should not be directly compared point-wise with theirs; the same caveat, of course, applies to **Table 8**. **Table 8** summarizes the reported performance of the most closely related methods. Because these studies use different datasets, object definitions, and evaluation protocols, the listed metrics are indicative rather than directly comparable.

**Table 8:** Reported performance of closely related sticky-trap pest detection studies. NR = not reported.

Method	Target scenario	Dataset scale	mAP@0.5	Model footprint	Inference strategy
YOLO-YSTs (Huang et al., 2025)	Pests on yellow sticky traps	NR	86.8%	NR	Single-pass, 139 FPS as reported
YOLOv11n-LMN (Meng et al., 2026)	Multi-class pests on sticky traps	NR	91.3%	Lightweight (PP-LCNet backbone)	SAHI slice-aided inference
YOLOv11n-WNE (this study)	Thrips on sticky traps	2, 400 images/40, 480 instances	91.5%	2.11M/4.5 MB	Single-pass, no slicing

### 4.5 Limitations and future work

Although the proposed method achieves 91.5% mAP@0.5 on the test set, it still has the following limitations: (1) the NWD normalization constant D needs to be set manually according to dataset characteristics, lacking an adaptive mechanism; (2) although the computational overhead of WSF is lower than that of transposed convolution, it still increases somewhat compared with nearest-neighbor interpolation; (3) currently only single-class thrips detection is performed, whereas sticky traps in real greenhouse environments often contain multiple pest species simultaneously (e.g., whiteflies, leaf miners); and (4) all training and test images contain thrips, and no dedicated negative samples consisting solely of background distractors (dust, non-target insects, or plant debris) were collected, so robustness against such distractors is assessed through the false-positive source analysis in Section 3.8 rather than through dedicated negative-sample evaluation.

Future work may explore the following directions: (1) design an adaptive NWD normalization scheme; (2) further investigate more lightweight wavelet-transform approximation algorithms; (3) extend the model to simultaneous multi-class pest detection and build a general greenhouse sticky-trap pest detection system; and (4) fuse time-series data and environmental variables to realize pest-population dynamic prediction and early warning.

## 5 Conclusion

Targeting the problems that thrips objects on sticky traps are extremely small, densely distributed, and insufficiently detected by traditional methods, this paper proposes a thrips detection method based on an improved YOLOv11n, named YOLOv11n-WNE. The main conclusions are as follows:

1. The designed WSF (Wavelet Unpooling StarFusion) network preserves spatial-detail information through WaveletUnPool wavelet-based upsampling and captures high-order interactions among multi-scale features through the StarFusion module, effectively enhancing the feature representation ability of small thrips objects. Experiments show that the WSF module improves mAP@0.5 from 87.4% to 91.2% and increases recall by 4.2 percentage points.
2. The introduced NWD (Normalized Wasserstein Distance) loss function provides a smooth gradient signal for small-object bounding-box regression through Gaussian-distribution modeling and Wasserstein-distance measurement. The combined NWD + CIoU loss improves mAP@0.5 to 91.0% and effectively alleviates the thrips missed-detection problem in dense scenarios.
3. The designed EfficientHead lightweight detection head reduces the detection-head parameter count by about 23% and the computation by about 16% through the G = 8 grouped-convolution strategy, with only a 0.3 percentage-point loss in mAP@0.5, endowing the model with edge-deployment potential.
4. The comprehensive YOLOv11n-WNE model with the three improvements achieves mAP@0.5 of 91.5%, precision of 88.2%, and recall of 85.0% on the 2, 400-image thrips dataset, with only 2.11M parameters and a model size of only 4.5 MB, and an inference speed of 123.8 FPS on the RTX 4090D GPU (14.5 FPS on the i7–11700 CPU), achieving the best accuracy–efficiency comprehensive performance among all compared models.

This method provides an efficient and lightweight technical solution for intelligent greenhouse thrips monitoring, and is of significant theoretical and practical value for promoting precise pest control in protected agriculture.

## Data Availability

The raw data supporting the conclusions of this article will be made available by the authors, without undue reservation.
